# Lyophilized particles and ethanolic extracts of Antrodia cinnamomea mycelia suppress the tumorigenicity of head and neck cancer cells in vivo

**DOI:** 10.7603/s40681-014-0026-x

**Published:** 2014-11-22

**Authors:** Ching-Wen Chang, Yu-Syuan Chen, Chien-Chih Chen, Chin-Chu Chen, Sen-Je Sheu, Ting-wei Lin, Te-Chang Lee, Jeng-Fan Lo

**Affiliations:** 1 Institute of Oral Biology, National Yang-Ming University, No. 155, Section 2, Li-Nong Street, 112 Taipei, Taiwan; 2 Department of Biotechnology, Hungkuang University, 433 Taichung, Taiwan; 3 Grape King Inc, 320 Taoyuan County, Taiwan; 4 Institute of Biomedical Sciences, Academia Sinica, 115 Taipei, Taiwan; 5 Graduate Institute of Chinese Medical Science and Institute of Medical Science, China Medical University, 404 Taichung, Taiwan; 6 Genome Research Center, National Yang-Ming University, 112 Taipei, Taiwan; 7 Department of Dentistry, Taipei Veterans General Hospital, 112 Taipei, Taiwan

**Keywords:** *Antrodia cinnamomea;*, Head and neck cancer

## Abstract

Head and neck cancer (HNC) is one of the most common forms of cancer in Taiwan. In addition, head and neck cancer cells (HNCs) are highly tumorigenic and resistant to conventional therapy. Therefore, development of new therapeutic regimens that are adjuvant to conventional treatments would benefit future head and neck cancer therapy. In this study, we found that the lyophilized particles and ethanolic extracts of *Antrodia cinnamomea* mycelia inhibited the tumor growth of HNCs by xenograft assay *in vivo*. Moreover, administration of lyophilized particles or ethanolic extracts to nude mice did not cause significant side effects. Our study revealed that the *Antrodia cinnamomea* mycelia extract (ACME) efficiently inhibited the tumorigenicity of HNCs without causing organ failure. Furthermore, it showed that ACME may work as a novel drug candidate for alternative treatments for head and neck cancer.

## 1. Introduction

Head and neck cancer (HNC) represents the sixth most common form of cancer with an estimated 600,000 new cases annually worldwide [1]. Head and neck squamous cell carcinoma (HNSCC) represents more than 95% of all head and neck cancers [2]. In spite of the many advances in our understanding in prevention and treatment of other types of cancers, the five-year survival rate after diagnosis for HNSCC remains low, at approximately 50% [3]. Due to a high recurrence, a high mortality rate, and a resistance to conventional therapies, the development of new chemopreventive agents for HNSCC that are effective on high risk populations (or patients) and that are adjuvant to conventional treatments is an important research priority.

*Antrodia cinnamomea*, also called *Antrodia camphorata*, a rare medical mushroom of the family Polyporaceae, mainly grows on the inner wood wall of *Cinnamomun kanehiraihay (Lauraceae)* in Taiwan [4]. In traditional Taiwanese medicine, the fruit bodies of *Antrodia cinnamomea* have been widely used to treat diarrhea, intoxication, hypertension, hepatoprotection, itchy skin [5], and cancer prevention [6]. However, the fruit bodies of *Antrodia cinnamomea* are rare and expensive, partially due to the difficulty in cultivation [6]. The submerged culture of *Antrodia cinnamomea* mycelia is one of the most effective methods for application in the formulation of nutraceuticals and functional foods [7]. The biological functions and activities of Antrodia cinnamomea mycelia extract (ACME) have been identified [6]. In our study, we found that YMGKI-1, one of the active components from ACME, can inhibit cancer-initiating cell properties through exaggerated autophagic cell death [8]. However, the anticancer effect of the crude ACME in HNSCC with animal models remains unclear.

In the present study, we examined the therapeutic effect of lyophilized particles and ethanolic extracts of *Antrodia cinnamomea* mycelia by xenograft assays. Our data showed that oral feeding with ACME reduced the tumor growth of HNSCC in tumor-bearing mice without causing organ failure. Thus, ACME may work as a novel drug candidate for alternative treatments for head and neck cancer.

**Fig. 1:**
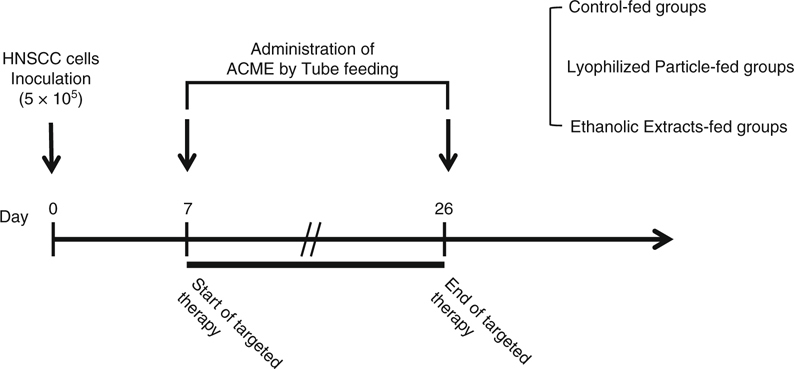
Overview of the xenograft mice model with ACM or ACME feeding procedure. Parental HNSCC cells (5 × 10^5^ cells) were subcutaneously implanted into the back of nude mice to develop tumor to a size about 0.1 cm^3^. At day 7 after cells implantation, tumor-bearing nude mice were fed with lyophilized particles or ethanolic extracts diet (3 times per week) for 21 day by tube feeding, respectively.

## 2. Materials and methods

Preparation of lyophilized particles and ethanolic extracts of *Antrodia cinnamomea* mycelia (ACM) were obtained from the Biotechnology Center, Grape King Inc., in Taoyuan County, Taiwan [7]. Matured mycelia were separated from the red-brown broth and then lyophilized, ground to a powder, and stored at room temperature [9]. Then, the lyophilized particles of *Antrodia cinnamomea* mycelia were used for this study. To prepare the ethanolic extracts of ACM, 1 gram of the above lyophilized particles was further extracted with 95% ethanol at 30°C for 24 h. The filtrates dissolved in 95% ethanol were dried under a vacuum to collect the ethanolic extracts of ACM [10].

### 2.1 Cell lines

SAS tongue carcinoma cells, human HNSCC cell lines, obtained from the Japanese Collection of Research Bioresources (Tokyo, Japan) were cultured in a DMEM medium containing 10% fetal bovine serum (Grand Island, NY) [11]. Cells were cultured at 37°C in a 5% CO_2_ environment. Short tandem repeat (STR) genotyping was performed for authentication of used cell lines by Genelabs Life Science Corporation (Taipei, Taiwan).

### 2.2 In vivo tumorigenic assay

All of the animal practices in this study were approved and were in accordance with the Institutional Animal Care and Use Committee (IACUC) of National Yang-Ming University, Taipei, Taiwan (IACUC approval nos. 1001223 and 991235). The antitumorigenic effect of lyophilized particles and ethanolic extracts was examined in 6-week-old nude BALB/c nu/nu mice (n = 4 per group). HNSCC cells (5 × 10^5^ cells) were subcutaneously injected into the back of the nude BALB/c mice (n = 4 per group). Tumors became palpable in about a week. Then, the lyophilized particles or ethanolic extracts were fed by tubing. Treatments were done on a schedule of three times per week for 21 days, after which tumor volumes were determined. The volume of the tumors was calculated via the following formula: (Length × Width2)/^2^ [12].

### 2.3 Statistics

An unpaired *t*-test was used for statistical analysis. The results were considered to be statistically different when the *P* value was <0.05.

## 3. Results

### 3.1 Anti-tumorigenic ability and side effects of ACM and ACME in tumor-bearing nude mice

**Fig. 2:**
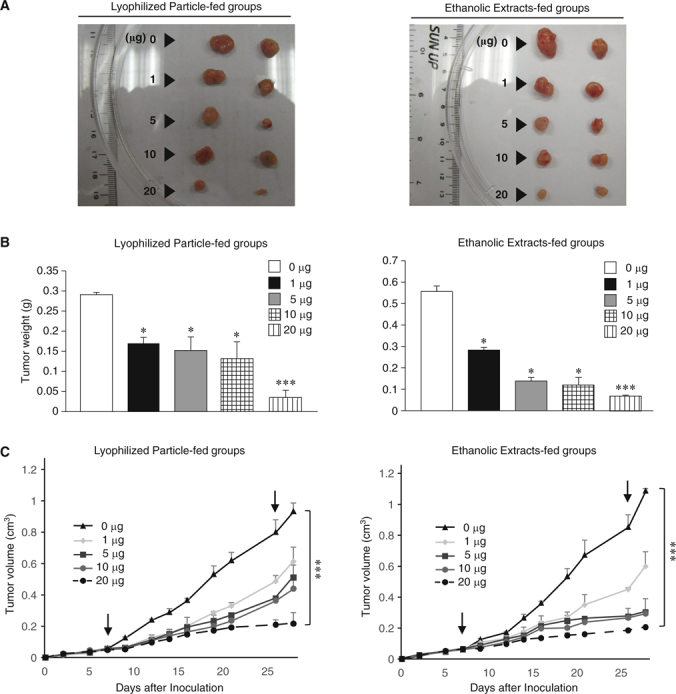
ACM or ACME feeding reduced the tumor growth in BALB/c mice injected with SAS cells. Mice were injected with SAS cells followed by feeding with ACM or ACME, and then sacrificed as described in Figure 1. (A) Images of dissected tumors were collected on day 28 from lyophilized particle-fed and ethanolic extracts-fed mice. (the first row: H_2_O (control), the second row: treated with 1 μg, the third row: treated with 5 μg, the fourth row: treated with 10 μg and the fifth row: treated with 20 μg). (B) The tumors were removed from lyophilized particle-fed and ethanolic extracts-fed mice and weighed. (C) The tumor growth curves of HNSCC cells in nude mice treated with lyophilized particle and ethanolic extracts were recorded.

**Fig. 3:**
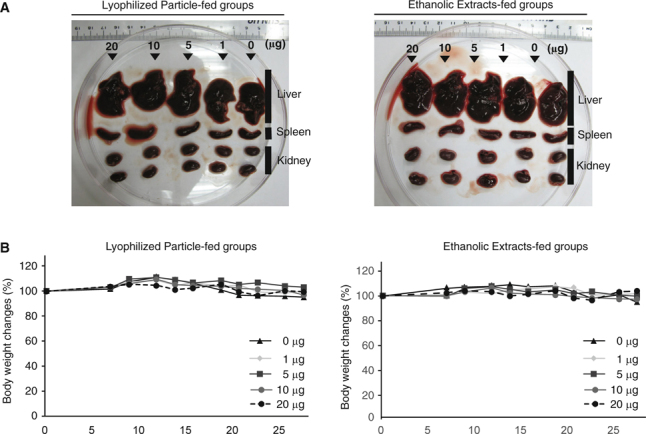
Gross appearance of organs and body weight measurement during the ACME feeding model. Gross appearance of organs of mice followed by lyophilized particles or ethanolic extracts treatment after 28 day of tumor development. (B) Measurement of body weight of tumor-bearing nude mice (n = 4) during the course of the lyophilized particles feeding or ethanolic extracts feeding.

*Antrodia cinnamomea* has been used for treatments of diseases and illnesses such as diarrhea, intoxication, hypertension, abdominal pain, itchy skin and some forms of cancer [13]. With this in mind, we wanted to determine if ACM and ACME treatment could attenuate the tumor growth of HNSCC *in vivo*. To investigate whether treatment of lyophilized particles and ethanolic extracts of Antrodia cinnamomea could exhibit anti-tumorigenic effects, BALB/c mice were inoculated with SAS cells. When the tumors became palpable, tumor-bearing nude mice were fed with either a lyophilized particles or an ethanolic extracts diet 3 times per week for 21 days by tube feeding, (Figure 1). Effectively, tumor-bearing mice receiving either the lyophilized particles or the ethanolic extracts treatment afterward displayed reduced tumor growth and tumor weight in comparison to that of the control mice (Figure 2A and 2B). As shown in Figure 2C, the mean tumor volume reached 1 cm^3^ in the control mice 4 weeks after tumor injection; in contrast, a significant suppression of tumor volume was observed in the mice that were tube fed a diet of either lyophilized particles or ethanolic extracts. The antitumorigenic ability of lyophilized particles was dose-dependent with an inhibition rate from 22.6% to 65.3%. In the ethanolic extractsfed group, the inhibition rate was from 18.9% to 54.9%. Intriguingly, neither the lyophilized particles nor the ethanolic extracts treatment caused significant side effects such as a change of gross appearance of organs or body weight in the tumor-bearing mice (Figure 3A and 3B).

## 4. Discussion

Accumulated evidence has suggested that *Antrodia cinnamomea* could be a potential agent for cancer therapy. For example, Yang et al found that the fermented culture broth of *Antrodia cinnamomea* promotes cell cycle arrest and apoptosis of breast cancer cells through suppression of the MAPK signaling pathway [14]. Recently, the anticancer effects of active compounds from *Antrodia cinnamomea* have been identified [6]. Yeh et al. demonstrated that a sesquiterpene lactone antrocin from Antrodia cinnamome inhibited cell proliferation in non-small-cell lung cancer cells [15]. Yeh et al. also demonstrated that a mixture of compounds from *Antrodia cinnamomea* showed a synergistic cytotoxic effect in HT-29 cells [16]. Moreover, in the case of Antrodia cinnamome, the anti-cancer efficacy may be attributed to multiple active compounds. But the molecular mechanism and active compounds also need to be studied.

Recent data have demonstrated that cancer cells are functionally heterogeneous and undergo not only proliferation but also differentiation and maturation to a certain degree [17]. Cancer initiating cells (CICs), a more resistant, self-renewing and malignant subpopulation of cancer cells, are considered a novel target in cancer therapy. Elimination of CICs apparently requires exhaustion of stemness and promoting differentiation by targeting self-renewal pathways. Thus, it has been reported that colorectal CICs/CSCs are induced differentiation and their response to chemotherapy can be increased by bone morphogenetic protein 4 (BMP-4) [18]. Moreover, resveratrol, abexinostat and curcumin were previously observed to impair CIC properties, induce CIC differentiation and reduce tumor malignancy through inhibiting self-renewal signaling pathways [19-21]. In our previous study, we demonstrated that HN-CICs possess stemness properties, which are characterized by up-regulation expression of selfrenewal gene Oct-4 and Nanog and differentiation ability [22]. Our previous findings found that YMGKI-1, one of the active components from ACME, can diminish tumorigenicity through the blocking of self-renewal ability and induction of CIC differentiation [8]. Together, these studies suggest Antrodia cinnamome possesses the ability to target CICs.

In the present study, the anti-tumor activities of lyophilized particles and ethanolic extracts of *Antrodia cinnamomea* mycelia were identified. We found that the tumor-bearing mice which were gavaged with up to 1~20 μg of *Antrodia cinnamomea* mycelium or its extracts three times per week had a reduction of tumor size but did not have organ damage (Figures 2 and 3). Our findings suggest that the product of *Antrodia cinnamomea* mycelia could be a promising adjuvant to conventional treatments for HNSCC that is effective in high risk populations (or patients).

## 5. Conflict of Interests

No potential conflict of interests was disclosed.

## 6. Authors’ Contributions

Ching-Wen Chang and Yu-Syuan Chen contributed equally to this article.

## 7. Acknowledgments

The authors thank Dr. K-W Chang (Department of Dentistry, National Yang-Ming University) for providing critical comments. This study was supported by research Grants from National Science Council (NSC99N024, NSC100-2314-B-040-001, NSC100N446, and NSC101N050), Taipei Veterans General Hospital (V99ER2-006 and VGHUST99-P6-39), National Yang-Ming University (Ministry of Education, Aim for the Top University Plan: 99ACT303-2, 100ACT513, 100ACT807, 101ACT513, and 102ACTC14), MacKay Hospital (Mackay 10187), and Grape King Inc. (YM99C021 and 101J041) in Taiwan.

## References

[1] Haddad RI, Shin DM. (2008). Recent advances in head and neck cancer. N Engl J Med.

[2] Jemal A, Siegel R, Ward E, Hao Y, Xu J (2008). Cancer statistics, 2008. CA Cancer J Clin.

[3] Siegel R, Naishadham D, Jemal A (2012). Cancer statistics, 2012. CA Cancer J Clin.

[4] Shen YC, Yang SW, Lin CS, Chen CH, Kuo YH (1997). Zhankuic acid F: a new metabolite from a formosan fungus Antrodia cinnamomea. Planta Med.

[5] Wu MD, Cheng MJ, Wang WY, Huang HC, Yuan GF (2011). Antioxidant activities of extracts and metabolites isolated from the fungus Antrodia cinnamomea. Nat Prod Res.

[6] Geethangili M, Tzeng YM. Review of Pharmacological Effects of Antrodia camphorata and Its Bioactive Compounds. Evid Based Complement Alternat Med 2011: 212641. 10.1093/ecam/nep108 PMC3095428 19687189

[7] Mau J-L, Huang P-N, Huang S-J, Chen C-C. (2004). Antioxidant properties of methanolic extracts from two kinds of Antrodia camphorata mycelia. Food Chemistry.

[8] Chang CW, Chen CC, Wu MJ, Chen YS, Chen CC, *et al*. Active Component of Antrodia cinnamomea Mycelia Targeting Head and Neck Cancer Initiating Cells through Exaggerated Autophagic Cell Death. Evid Based Complement Alternat Med 2013: 946451. 10.1155/2013/946451 PMC3703405 23843890

[9] Chen TI, Chen CW, Lin TW, Wang DS, Chen CC (2011). Developmental toxicity assessment of medicinal mushroom Antrodia cinnamomea T.T. Chang et W.N. Chou (higher Basidiomycetes) submerged culture mycelium in rats. Int J Med Mushrooms.

[10] Chen YS, Pan JH, Chiang BH, Lu FJ, Sheen LY (2008). Ethanolic extracts of Antrodia cinnamomea mycelia fermented at varied times and scales have differential effects on hepatoma cells and normal primary hepatocytes. J Food Sci.

[11] Okumura K, Konishi A, Tanaka M, Kanazawa M, Kogawa K (1996). Establishment of high- and low-invasion clones derived for a human tongue squamous-cell carcinoma cell line SAS. J Cancer Res Clin Oncol.

[12] Chang CW, Chen YS, Chou SH, Han CL, Chen YJ, *et al*. Distinct Subpopulations of Head and Neck Cancer Cells with Different Levels of Intracellular Reactive Oxygen Species Exhibit Diverse Stemness, Proliferation, and Chemosensitivity. Cancer Res. 2014. 10.1158/0008-5472.CAN-14-0626 25217518

[13] Kuo MC, Chang CY, Cheng TL, Wu MJ. (2008). Immunomodulatory effect of Antrodia camphorata mycelia and culture filtrate. J Ethnopharmacol.

[14] Yang HL, Kuo YH, Tsai CT, Huang YT, Chen SC (2011). Anti-metastatic activities of Antrodia camphorata against human breast cancer cells mediated through suppression of the MAPK signaling pathway. Food Chem Toxicol.

[15] Yeh CT, Huang WC, Rao YK, Ye M, Lee WH (2013). A sesquiterpene lactone antrocin from Antrodia camphorata negatively modulates JAK2/STAT3 signaling via microRNA let-7c and induces apoptosis in lung cancer cells. Carcinogenesis.

[16] Yeh CT, Rao YK, Yao CJ, Yeh CF, Li CH (2009). Cytotoxic triterpenes from Antrodia camphorata and their mode of action in HT-29 human colon cancer cells. Cancer Lett.

[17] Jordan CT, Guzman ML, Noble M. (2006). Cancer stem cells. N Engl J Med.

[18] Lombardo Y, Scopelliti A, Cammareri P, Todaro M, Iovino F (2011). Bone morphogenetic protein 4 induces differentiation of colorectal cancer stem cells and increases their response to chemotherapy in mice. Gastroenterology.

[19] Hu FW, Tsai LL, Yu CH, Chen PN, Chou MY (2012). Impairment of tumor-initiating stem-like property and reversal of epithelial-mesenchymal transdifferentiation in head and neck cancer by resveratrol treatment. Mol Nutr Food Res.

[20] Salvador MA, Wicinski J, Cabaud O, Toiron Y, Finetti P (2013). The histone deacetylase inhibitor abexinostat induces cancer stem cells differentiation in breast cancer with low xist expression. Clin Cancer Res.

[21] Zhuang W, Long L, Zheng B, Ji W, Yang N (2012). Curcumin promotes differentiation of glioma-initiating cells by inducing autophagy. Cancer Sci.

[22] Chiou SH, Yu CC, Huang CY, Lin SC, Liu CJ (2008). Positive correlations of Oct-4 and Nanog in oral cancer stem-like cells and highgrade oral squamous cell carcinoma. Clin Cancer Res.

